# Multiomics‐based study of amniotic fluid small extracellular vesicles identified Moesin as a biomarker for antenatal hydronephrosis

**DOI:** 10.1002/ctm2.1360

**Published:** 2023-08-02

**Authors:** Jingzhi Li, Ying Fu, Qiaoshu Liu, Kuifang Shen, Ruojin Yao, Yimei Fu, Yang Lu, Mingkun Xie, Wenyan Jian, Ming Guo, Lei Dai, Weishe Zhang

**Affiliations:** ^1^ Department of Obstetrics Xiangya Hospital Central South University Changsha China; ^2^ Hunan Engineering Research Center of Early Life Development and Disease Prevention Changsha China; ^3^ Department of Oncology, NHC Key Laboratory of Cancer Proteomics and State Local Joint Engineering Laboratroy for Anticancer Drugs Xiangya Hospital, Central South University Changsha China

Dear Editor,

Antenatal hydronephrosis (ANH) is the most common congenital anomaly of the urinary tract. Pregnancy outcome and fetal prognosis are closely related to the severity of ANH.^1^ Approximately 15%−20% of children with ANH result in postnatal renal obstruction, which will lead to rapid deterioration of renal function.^2^ Parameters measured by prenatal ultrasonography (US), especially fetal anteroposterior renal pelvic diameter (APD/APRPD), are used as the most predictive indicators to detect and diagnose ANH.^3^ However, US tend to be progressive and inconsistent, even lack of diagnostic criteria to evaluate renal function and its obstruction after birth. These highlight the imperative to identify biologic diagnostic marker for ANH. In this study, we extracted small extracellular vesicles riched samples (sEVs) from supernatant amniotic fluid (AF), identified and verified the high expressed Moesin as an effective biomarker for ANH diagnosis.

We recruited 37 pregnant women with ANH in different grades and 28 normal pregnant women with high risks of age over 35 or serum screening (Table S1). Routine amniocentesis was performed during the middle of pregnancy and the chromosome abnormalities of fetal were excluded. The 65 samples were divided into two cohorts: 6 samples including severe ANH (*n* = 3) and normal cases (*n* = 3), were used as the testing set for biomarker discovery. The other 59 samples (34 ANH vs. 25 normal cases), were used as the validation set. The clinical information of the samples is summarized in Table S2. The sEVs in supernatant AF were extracted using size‐exclusion chromatography with ultracentrifugation,^4^ then identified by characteristics^5^ (Figure 1). Label‐free proteomic and mRNA sequencing were used to identify proteins and mRNAs of sEVs and cells in AF, respectively (see Figure S1 for workflow).

**FIGURE 1:**
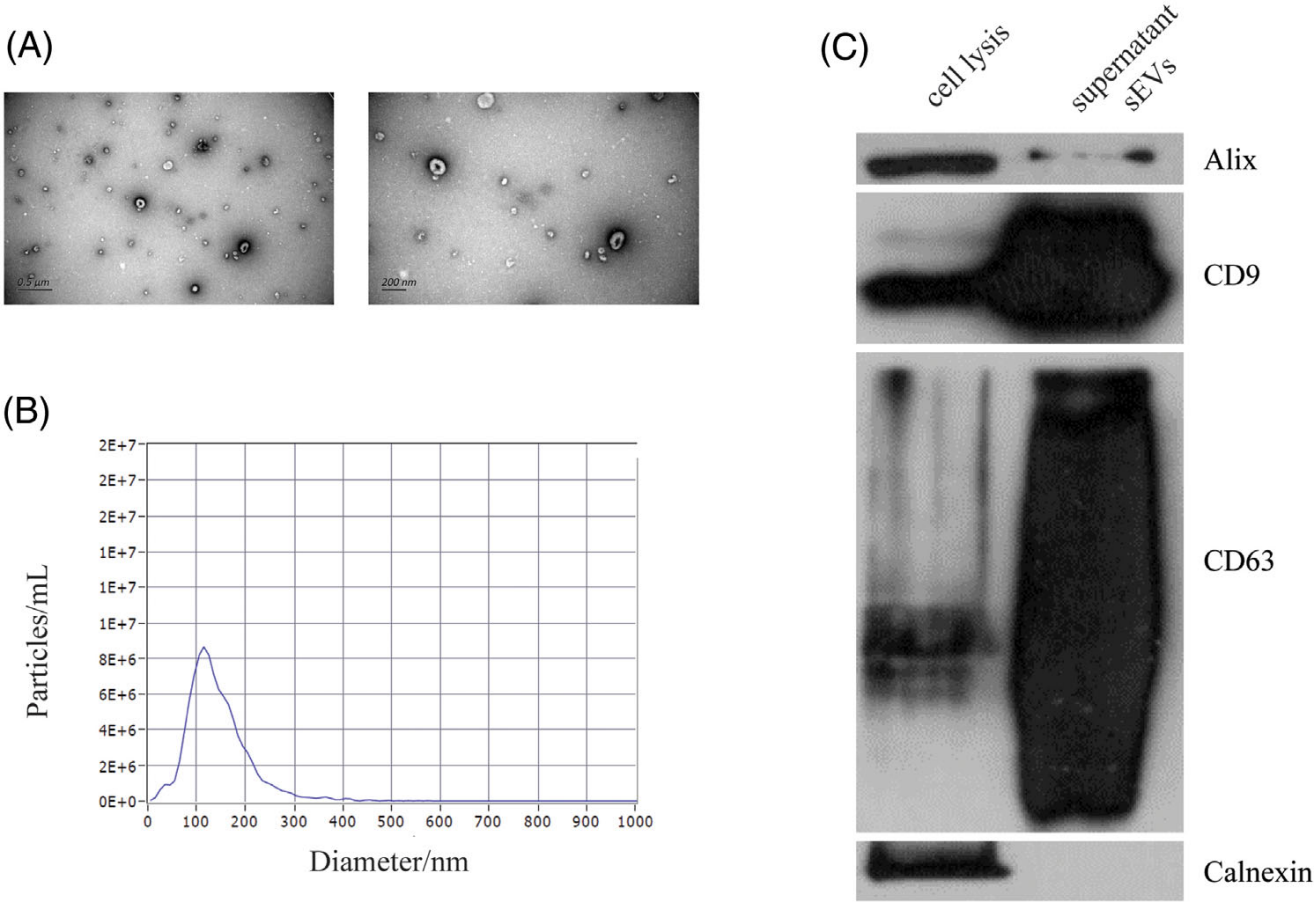
Verification of the identity of sEVs isolated from amniotic fluid. (A) Transmission electron microscopy (TEM) images of sEVs combined with SEC and UC. (B) NTA detection of sEVs enriched from AF, approximately 75‐200 nm in diameter. (C) EV markers CD9, CD63 and Alix detection in the sEVs isolated from amniotic fluid, and Calnexin, a negative marker of EV, was absent in our isolated sEVs.

Sample clustering and principal component analysis (PCA) indicated a relatively well stability with high identity in each group (Figure 2A and Figure S2A) and obvious differences between groups (Figure 2B and Figure S2B). A total of 1308 proteins and 4118 mRNAs were identified (Figure 2C and Figure S2C). We depicted a draft map of mRNA and protein expressions in sEVs and cells from normal AF for the first time. In sEVs versus cells groups, 1128 proteins (Figure 2D, E) and 3047 mRNAs (Figure S2D, E) were found to be differentially expressed through differential expressed genes (DEGs) analysis, different gene profiles were demonstrated by Gene Ontology (GO) (Figure 2F and Figure S2F) and Kyoto Encyclopedia of Genes and Genomes (KEGG) analyses (Figure 2G and Figure S2G).

**FIGURE 2:**
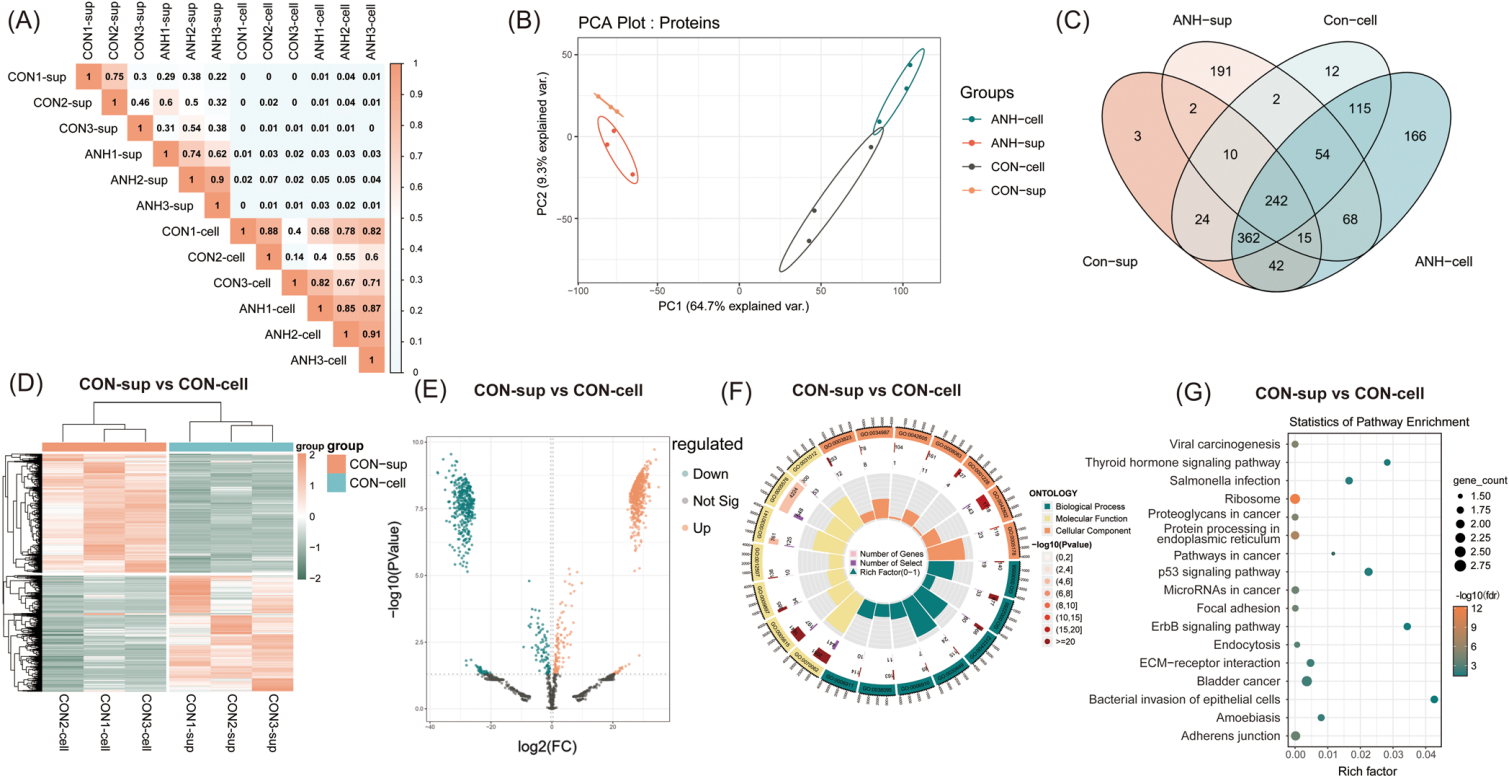
Target analysis of differentially expressed proteins between sEVs and cells in normal fetal groups. (A) Corrplot correlation analysis of within‐group identity and between‐group difference of samples confirmed by MS analysis. (B) Principal component analysis (PCA) of proteins that separated the four groups: CON‐sup, ANH‐sup, CON‐cell, and ANH‐cell. (C) Venn diagram of proteins differentially expressed between the four groups. A total of 1308 proteins were identified. Among these, 242 (18.5%) proteins were present in all samples. (D) Heatmap of proteins differentially expressed between CON‐sup and CON‐cell. 1128 proteins were found to be differentially expressed in sEVs versus cells, including 636 upregulated and 492 downregulated proteins. E) Volcano plots of statistical analysis of all proteins identified in CON‐sup and CON‐cell. F) GO analysis of proteins differentially present in CON‐sup versus CON‐cell through circus plot. (Presenting the main 6 of Biological Process, Cellular Component and Molecular Function, respectively.) G) Bubble plot showing the numbers of DEG‐targeted proteins in each KEGG pathway (targeted genes > 10). CON‐sup, normal fetal amniotic fluid sEVs; ANH‐sup, ANH amniotic fluid sEVs; CON‐cell, normal fetal amniotic fluid cells; ANH‐cell, ANH amniotic fluid cells.

To further explore the special expression pattern in sEVs of ANH, we compared the proteins and mRNAs in sEVs between ANH and normal samples, respectively. We identified 116 differential expression proteins (Figure 3A, B; Tables S3) and 836 differential expression mRNAs (Figure 3E, F, TableS4) between ANH amniotic fluid sEVs (ANH‐sup) and normal fetal amniotic fluid sEVs (CON‐sup). GO and KEGG analysis indicated that DEGs were mainly in extracellular localization and associated with development (Figure 3C,G) even enriched in tissue morphogenesis, especially renal development, such as developmental process (GO:0032502) and kidney development (GO:0001822) (Figure 3D,H).

**FIGURE 3:**
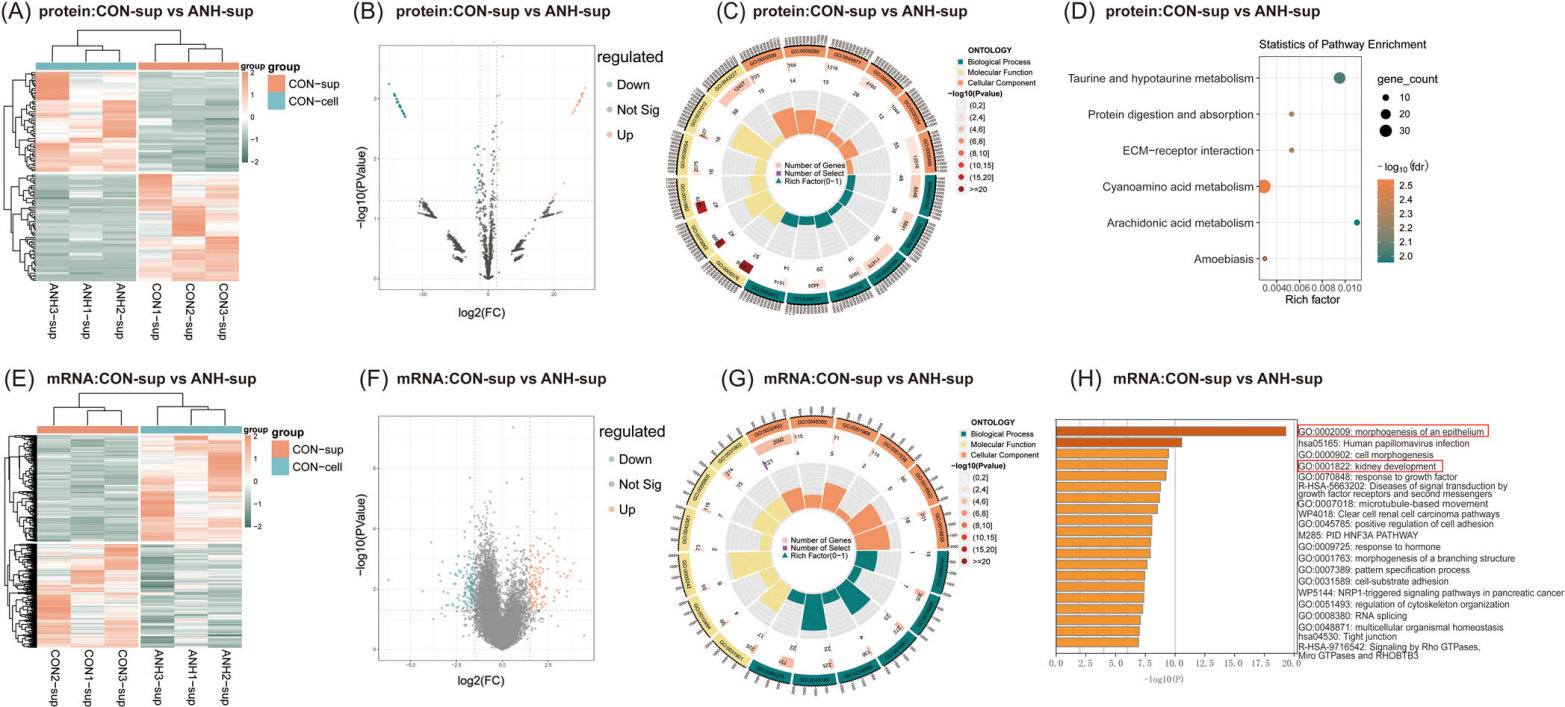
Target analysis of differentially expressed genes between the ANH and normal sEVs groups. (A) Heatmap of proteins differentially expressed between CON‐sup and ANH‐sup. One hundred sixteen differential expression proteins were identified between ANH‐sup and CON‐sup, including 56 up‐regulated and 60 downregulated proteins. (B) Volcano plots of statistical analysis of all proteins identified in CON‐sup and ANH‐sup. (C) GO analysis of proteins differentially present in CON‐sup versus ANH‐sup by circus plot. (Presenting the main 6 of biological process, cellular component and molecular function, respectively). (D) Bubble plot showing the numbers of DEG‐targeted proteins in each KEGG pathway. (E) Heatmap of mRNAs differentially expressed between CON‐sup and ANH‐sup. Eight hundred thirty‐six differential expression mRNAs were identified between ANH‐sup and CON‐sup, including 421 upregulated and 415 down‐regulated mRNAs. (F) Volcano plots of statistical analysis of all mRNAs identified in CON‐sup and ANH‐sup. (G) Circus plot showing GO analysis of mRNAs differentially present in CON‐sup versus ANH‐sup. (Presenting the main 5 of biological process, cellular component and molecular function, respectively). (H) The top 20 KEGG pathways of mRNAs targeted by the DEMs identified between CON‐sup and ANH‐sup. CON‐sup, normal fetal amniotic fluid sEVs; ANH‐sup, ANH amniotic fluid sEVs. CON‐sup, normal fetal amniotic fluid sEVs; ANH‐sup, ANH amniotic fluid sEVs.

Furthermore, 18 differentially expressed genes with consistent expression trends of proteins and mRNAs in DEGs were eventually identified, including 10 up‐regulated and 8 down‐regulated genes (Table S5). GO, KEGG, and Protein‐Protein Interaction Networks analysis indicated that they might be involved in extracellular matrix (ECM) (Figure 4A,B). The mRNA and protein expression fold change ratios from 18 candidate genes were significant positive correlation (Figure 4C). To investigate the potential biomarkers specific in sEVs, Moesin was identified as the unique up‐regulated expressed in sEVs of ANH but absent in cells (Figure 4D,E). As an Ezrin‐Radixin‐Moesin protein, Moesin was phosphorylated by transforming growth factor (TGF)‐β1 to promote epithelial to mesenchymal transition involving in renal fibrosis through Erk signaling pathway.^6^ It was also high expressed in obstructive or injured kidneys of three chips downloaded from gene expression omnibus (GEO) (GSE48041, GSE42303) and ArrayExpress (E‐MTAB‐6640) databases (Figure S3A).

**FIGURE 4:**
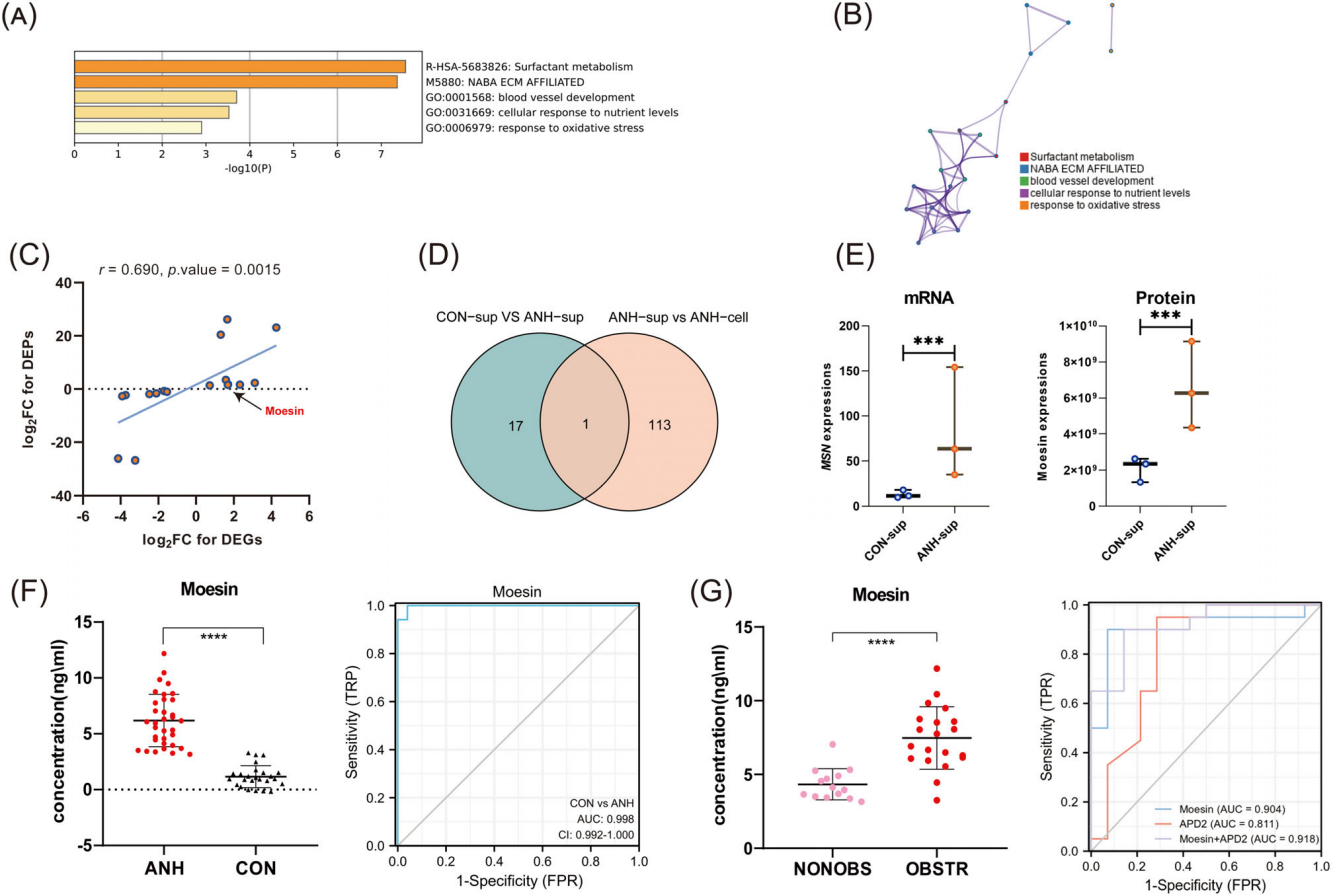
Identification and verification of Moesin as an effective biomarker for ANH diagnosis. (A) Bar chart of clustered enrichment ontology categories (GO and KEGG terms) analysis by Metascape of 18 candidate genes. (B) PPI analysis of 18 candidate genes. Each term is represented by a circle node, the size of which is proportional to the number of input genes that fall under that term, and the color of which represents the cluster identity (i.e., nodes of the same color belong to the same cluster). An edge connects terms with a similarity score greater than 0.3 (the thickness of the edge represents the similarity score). (C) Scatter plot of the pearson correlation between mRNA and protein expression FC ratios from 18 candidate genes. (D) Venn plot for the candidate Moesin that specifically enriched in ANH‐sup by intersecting two groups (CON‐sup vs. ANH‐sup and ANH‐sup vs. ANH‐cell). (E) Significant up‐regulated Moesin expression level in ANH of sEVs. (F) High‐expressed Moesin and ROC curve analysis in fetuses with ANHs compared with normal fetuses (34 ANH vs. 25 normal cases). (G) High‐expressed Moesin and ROC curve analysis in OBSTR groups compared with NONOBS groups (20 OBSTR vs. 14 NONOBS cases). Significant (***p < 0.001, ****p < 0.0001); CON‐sup, normal fetal amniotic fluid sEVs; ANH‐sup, ANH amniotic fluid sEVs; CON‐cell, normal fetal amniotic fluid cells; ANH‐cell, ANH amniotic fluid cells; AUC, area under the curve; ROC, receiver operating characteristic; CON, normal fetuses; OBSTR, postnatal obstructive; NONOBS, postnatal nonobstructive.

To evaluate the possibility of Moesin serving as an ANH diagnostic biomarker, Moesin expression was examined by enzyme‐linked immunosorbent assay (ELISA) in validation set. Moesin was demostrated highly expressed in ANH (6.183 [interquartile ranges, IQR, 4.048–8.049]) compared with normal (1.161 [IQR, 0.464–1.511]) fetuses (****p < 0.0001). The expression of Moesin in sEVs could clearly discriminate ANH from normal fetuses (area under the curve [AUC]: 0.998 [95% confidence interval, 0.992 to 1] and *p* < 0.0001). The Moesin cutoff for predicting ANH was 3.131 ng/ml (sensitivity:100%, specificity:96%, positive predictive value (PPV):97%, negative predictive value (NPV):100%) (Figure 4F). Moesin expression was further validated higher in ANH than normal fetuses by qPCR in 12 samples (Figure S3B). Cross‐section measurements of APD are the most commonly used parameter to assess ANH in utero.^7^, ^8^ The 2nd trimester with APD over 4 mm and 3rd trimester with APD over 7 mm are general standard of ANH diagnosis for prenatal US^1^ and ANH with an APD over 15 mm is considered severe or significant.^9^ But the cutoff varied widely in different studies.^1^, ^10^ As the selective predictor of postnatal renal function,^3^ the single measurement standard APD showed some drawbacks and limitations. We performed an in‐depth analysis combining the ELISA value of Moesin with prenatal US records and outcome of postnatal renal obstruction. Moesin performed superior to APD in ANH diagnosis and postnatal renal obstruction prediction. The median values of Moesin were 7.477 (IQR, 6.100–8.718) for obstructive infants and 4.335 (IQR, 3.483–4.998) for nonobstructive infants in ANH (****p < 0.0001), with an optimal cutoff of 5.435 ng/ml and the AUC of 0.904, the sensitivity and specificity are all above 90%. Compared with APD, the specificity and NPV were greatly improved. In particular, when Moesin was combined with APD of the 2nd trimester, the AUC up to 0.918, achieving the best prediction of infant obstruction (Figure 4G and Figure S3C).

Collectively, our data indicate a specific proteomic and mRNA profiles of AF, and the elevated expression of Moesin in sEVs of AF could serve as a diagnostic marker for ANH. These findings open up a variety of future diagnostic option for ANH.

## CONFLICT OF INTEREST STATEMENT

The authors have declared that no competing interest exists.

## Supporting information

Supporting Information

## Data Availability

The datasets presented in this study can be found in online repositories. The raw data of transcriptomics study are available at https://dataview.ncbi.nlm.nih.gov/object/PRJNA838478?reviewer = p6j1fp8an4sqvh74htdgusc5r0. The raw data of proteomics study are available via ProteomeXchange with identifier PXD033988.
