# Supplementary material for: Multiomics‐based study of amniotic fluid small extracellular vesicles identified Moesin as a biomarker for antenatal hydronephrosis
Source: Clin Transl Med. 2023 Aug 2;13(8):e1360. doi: 10.1002/ctm2.1360 (PMC10395771; doi:10.1002/ctm2.1360)

**Supplemental materials**

**This article contains the following supplemental materials:**

**MATERIALS and METHODS**

**Supplemental Table 1.** ANH pregnant women demographics and characteristics.

**Supplemental Table 2.** Detailed clinical characteristics, follow-ups, and ELISA results of every ANH pregnant woman in our research (n=34).

**Supplemental Table 3.** List of all significant proteins identified between ANH-sup and CON-sup using MS. (P value<0.05, |log_2_FC|>1)

**Supplemental Table 4.** List of all significant mRNAs identified between ANH-sup and CON-sup using RNA sequencing. (P value<0.05, |log_2_FC|>1)

**Supplemental Table 5.** Genes and proteins with consistent differential expression trends after combined proteomics and mRNA sequencing analysis.

**Supplemental Figure 1.** Schematic workflow showing the label-free quantitative proteomic analysis and mRNA sequencing of sEVs and cells.

**Supplemental Figure 2.** Target analysis of differentially expressed mRNAs between sEVs and cells in normal fetal groups. (A) Corrplot correlation analysis that within-group identity and between-group difference of samples confirmed by RNA sequencing. (B) Principal component analysis (PCA) of mRNA that separated the four groups: CON-sup, ANH-sup, CON-cell, and ANH-cell. (C) Venn diagram of mRNAs differentially expressed between the four groups. A total 4,118 mRNAs were identified. Among these, 3,819 (93.6%) mRNAs were present in all samples. (D) Heatmap of mRNAs differentially expressed between CON-sup and CON-cell. 3,047 mRNAs were found to be differentially expressed in sEVs versus cells, including 1,483 upregulated and 1,564 downregulated mRNAs. (E) Volcano plot of statistical analysis of mRNAs identified in CON-sup and CON-cell. (F) GO analysis of mRNAs differentially present in CON-sup versus CON-cell through circus plot. (per 6 of Biological Process, Cellular Component and Molecular Function, respectively.) (G) Bar plot showing the numbers of DEG-targeted mRNAs in each KEGG pathway (targeted genes >10). CON-sup, normal fetal amniotic fluid sEVs; ANH-sup, ANH amniotic fluid sEVs; CON-cell, normal fetal amniotic fluid cells; ANH-cell, ANH amniotic fluid cells.

**Supplemental Figure 3.** Validation the expression of candidate Moesin as biomarker. (A) Exploration of Moesin expression in GEO (GSE48041, GSE42303) and ArrayExpress (E-MTAB-6640) databases. Moesin was found to increase with pathological progression of GSE48041 (log_2_FC= 2.858, P value = 1.04e-02, adjusted P value = 0.114), GSE42303 (log_2_FC= 1.226, P value = 6.57e-04, adjusted P value = 0.0108) and E-MTAB-6640 (log_2_FC = 1.295, P value = 1.543e-64, adjusted P value = 0.114). (B) Expression of candidate Moesin mRNA in sEVs in the validation cohort. The mean Moesin mRNA level in ANH patients (n=6) was higher than that in normal fetuses (n=6) (p=0.0128). Significant (*P<0.05). (C) The abilities of Moesin, and APD of different trimesters as biomarkers of ANH. Moesin distinguished the US grade from normal in fetuses. (n=25 normal fetuses, n=34 ANHs. Among ANHs, n=15 fetuses with SFU III-IV; n=10 fetuses with SFU II; n=9 fetuses with SFU I). ROC curves for the Moesin, APD2, and APD3 prediction of infant obstructions. Significant (**P<0.01, ****P<0.0001). AUC, area under the curve; ROC, receiver operating characteristic; CON, normal fetuses.

**MATERIALS and METHODS**

**Sample Handling**

The acquired AF samples were centrifuged at 3,000×g for 15 min at 4 °C, and the AF cell precipitate was retained. To minimize contamination of platelets, the supernatant was transferred to new tubes and centrifuged at 3,000×g for 15 min at 4 °C once more. The supernatant was filtered through a 0.22 μm filter. Paired AF cells and supernatants were stored at -80 °C until isolation and identification.

**Isolation of sEVs by Size-exclusion Chromatography (SEC) and Ultracentrifugation (UC) methods**

Ten-milliliter AF supernatant samples were thawed at 4 °C overnight. Then, the thawed samples were concentrated by a 50 mL 100 KD ultrafiltration tube (1:100) and centrifuged at 4,000×g for 15 min. One milliliter of condensed AF supernatant was loaded into a Sepharose-based CL-2B column (Echo9103A-10 mL; ECHO BIOTECH, China), which was prewashed with more than 20 mL of sterile PBS in advance. When no liquid was observed flowing out of the bottom of the columns after addition of a complete sample into the columns, PBS was used to elute sEVs and other fractions. Each 500 µL of effluent represented one fraction. A 100 KD ultrafiltration tube was used to further purify the 4 to 7 fractions collected. We collected the enriched sEVs into a tube and centrifuged them for 15 minutes at 4,000×g to enrich them.

**Nanoparticle Tracking Analysis (NTA)**

The vesicle-enriched suspension should be stabilized at concentrations between 1x10^7^/mL and 1x10^9^/mL. It was examined by a ZetaView PMX 110 instrument (Particle Metrix, Meerbusch, Germany) equipped with a 405 nm laser, which determines the size and quantity of particles isolated. An analysis of particle movement was conducted using NTA software using a video captured with a frame rate of 30 frames/second over a duration of 60 seconds (ZetaView 8.02.28).

**Transmission Electron Microscopy (TEM)**

We incubated 20 µL enriched sEVs on a copper mesh for 10 minutes at room temperature. After washing with sterile distilled water, the sEV-enriched fraction was stained with uranyl oxalate solution for 1 min and then allowed to dry under an incandescent lamp for 2 min. Imaging was observed and photographed under a transmission electron microscope (JEOL-JEM1400, Tokyo, Japan).

**Western Blot Analysis (WB)**

The protein of the enriched sEVs was denatured in 5× sodium dodecyl sulfonate (SDS) loading buffer and subjected to western blot analysis (10% SDS-polyacrylamide gel electrophoresis; 50 μg protein/lane). The detection antibodies were as follows: CD63 (sc-5275, Santa Cruz, CA, USA), TSG101 (sc-13,611, Santa Cruz, CA, USA), Alix (sc-53,540, Santa Cruz, CA, USA) and calnexin (10,427–2-AP, Promega, Madison, WI). The results were visualized on a Tanon 4600 automatic chemiluminescence image analysis system (Tanon, Shanghai, China).

**sEV Protein Quantification and Mass Spectrometry (MS) Analysis**

Following the manufacturer's instructions, the protein concentration of the enriched sEVs was quantified using a Pierce BCA Protein Assay Kit (Thermo Scientific, Product No. 23,225). Assays were performed by pipetting 10 µL of the standard and sEV-enriched fraction samples into a 96-well plate, adding 200 µL of the working reagent to each well, and thoroughly mixing every well. The plate was then incubated at 37 °C for 30 min, and the absorbance was measured at 562 nm. sEV samples were analyzed using a standard curve to determine their protein concentration. The sEV-associated protein concentration was more than 300 μg per mL.

In the experimental group, the 6-sample lysates, including severe ANH cases (n=3) and normal cases (n=3), were subjected to trypsin digestion and label-free quantitative proteomics analysis. Each sample was mixed with 3 milliliters of 1 mg/mL trypsin and 500 milliliters of 100 mM TEAB buffer and digested at 37 °C overnight. An equal volume of 1% formic acid (FA) was added to the digested proteins, and the mixture was centrifuged at 12,000×g for 5 min at room temperature. After loading the supernatant onto a C18 desalting column, it was washed three times with one mL of washing solution (0.1% FA, 4% acetonitrile) and then eluted twice with 0.4 mL of elution buffer (0.1% FA, 75% acetonitrile). The eluents were combined and freeze-dried by vacuum centrifugation. The lyophilized peptides were dissolved in 10 μL 0.1% FA and then injected into a homemade C18 Nano-Trap column (2 cm × 75 μm, 3 μm). Peptides were separated in a homemade analytical column (15 cm × 150 μm, 1.9 μm) with mobile phase A (0.1% FA in water) and mobile phase B (0.1% FA in 80% acetonitrile) at a flow rate of 600 nL/min for 60 min. The separated peptides were analyzed on a Q Exactive HF-X mass spectrometer (Thermo Fisher) with a Nanospray Flex™ (ESI) ion source and a spray voltage of 2.3 kV. The raw MS files were searched on the human UniProt database (http://www.UniProt.org). Carbamidomethyl was specified as a fixed modification. Oxidation of methionine and N-terminal acetylation were specified as variable modifications. For protein identification, at least one unique peptide was identified in the protein with a false discovery rate (FDR) obtained by Benjamini-Hochberg method of less than 0.01.

**RNA Isolation and Sequencing**

Total RNA was extracted and purified from sEV-enriched fractions using a miRNeasy® Mini kit (Qiagen, cat. No. 217,004) according to the manufacturer’s instructions. Agarose gels (1.5%) were used to monitor the degradation and contamination of RNA, especially DNA. Utilizing a NanoDrop 2000 spectrophotometer (Thermo Fisher Scientific, Wilmington, DE, USA), we measured the concentration and purity of RNA. RNA integrity was analyzed using an Agilent Bioanalyzer 2100 system (Agilent Technologies, Santa Clara, CA, USA) and an RNA Nano 6000 assay kit.

In the experimental group, the 6 RNA samples, including severe ANH cases (n=3) and normal cases (n=3), underwent library preparation and sequencing. A total of 5 ng of RNA per sample was used in the Ribo-ZeroTM Magnetic kit (Epicenter, Madison, WI, USA) to remove rRNA. The sequencing libraries were generated on an Ovation mRNA sequencing system (NuGEN, Redwood City, CA, USA) following the manufacturer's instructions, and index codes were used to attribute sequences to samples. The RNA samples were ligated with barcodes containing unique adaptor sequences to allow pooling of samples. The pooled DNA library elution was then processed with a NovaSeq 6000 platform for cluster generation and sequencing. The quality of the libraries was assessed using a Qubit fluorometer (Thermo Fisher Scientific, MA, USA). For each sEV sample, no less than 65 M clean reads were generated, and the Q30 percentage was no less than 87.25% for sequencing. We filtered raw reads using fastQC and aligned them to the GRCh38 human genome assembly by using HISAT2. From GENCODE (v.25), we retrieved annotations of mRNA in the human genome. The mRNAs were quantified and analyzed by DESeq2 R package and StringTie 1.3., respectively. We calculated the number of expressible mRNAs differentially expressed between the two groups using fpkm >= 0.05. Measurements of P values < 0.05 and |log2FC| > 1 are considered significant.

**Gene Ontology (GO) and Kyoto Encyclopedia of Genes and Genomes (KEGG) Analysis**

Combining the mRNA and protein results, by implementing the clusterProfiler R package, we analyzed the GO enrichment of differentially expressed genes (DEGs). In enrichment analysis, hypergeometric tests were used to identify GO items that were significantly enriched compared with the whole genome. GO analysis was performed using Metascape(https://metascape.org/). Meanwhile, the clusterProfiler R package was used to find KEGG pathways that were significantly enriched compared to the entire genome background. The statistical enrichment of differentially expressed genes in KEGG pathways was examined using KOBAS.

**qPCR Analysis**

In the validation group, RNA samples from ANH cases (n=34) and normal cases (n=25) were reverse transcribed into complementary DNA (cDNA) using HiScript Reverse Transcriptase (Vazyme; cat #R101-01) according to the manufacturer's instructions. The cDNA was PCR-processed using ChamQ Universal SYBR qPCR Master Mix (Vazyme; cat #Q711-02). Real-time quantitative PCR (qPCR) was performed on a Roche LightCycler 480 II instrument. qPCR was performed with the following procedure: 37 cycles at 94 °C for 15 s and 62 °C for 7 min. The primers used in this study were as follows: Moesin-F: tgtaaaccagagagctgctgg, Moesin-R: gaagagcacacatgagacagagaa; β-actin-F: tgacgtggacatccgcaaag, β-actin-R: ctggaaggtggacagcgagg. Ct values for each sample were determined and normalized to β-actin. The 2-^ΔΔC(t)^ equation was used to calculate the data in terms of relative expression.

**Enzyme-linked Immunosorbent Assay (ELISA)**

In the validation phase, sEV-derived Moesin expression was detected in ANH cases (n=34) and normal cases (n=25) by ELISA kits (Aviva; cat #okdd00404) according to the manufacturer’s instructions.

**Statistical Analysis**

We used plyr and reshape2 to sort and restructure the MS and sequence datas. Plots were generated using corrplot (0.92), ggbiplot (0.55), ggvenn (0.1.9), ggplot2 (3.3.6), pheatmap (1.0.12), circlize (0.4.15) or ComplexHeatmap (1.10.2). Statistical tests of MS and sequencing were performed using R 3.5.1 (www.r-project.org). P values < 0.05 and |log2FC| > 1 indicate differentially expressed mRNAs and proteins. qPCR and ELISA data were analyzed using GraphPad Prism 8.0. Data in all figures are expressed as the mean ± standard deviation (SD). Wilcoxon rank-sum tests was used for group comparisons. The diagnostic accuracy of the candidate genes was assessed with ROC curve analysis, as well as the area under the ROC curve (AUC) with SPSS 23.0 software (IBM, Ehningen, Germany). A significance level of P<0.05 was considered.

**Supplemental Table 1. ANH pregnant women demographics and characteristics (n=65).**

| **Variables** | | **Grading system (n=37)**  **for the first prenatal US** | | | **Normal(n=28)** |
| --- | --- | --- | --- | --- | --- |
|  |  | **SFU III-IV** | **SFU II** | **SFU I** |  |
| Sample size (%) | | 18(48.6) | 10(27.0) | 9(24.3) | 28 |
| Mean age of pregnant women (SD) | | 29.80(3) | 29.00(4) | 33.11(2.9) | 30(2.1) |
| Amniocentesis week (SD) | | 26.33(4.6) | 25.73(3.3) | 25.00(2.6) | 23(2.5) |
| Fetal gender (%) | Male | 12(66.7) | 7(63.6) | 6(66.7) | 13(52) |
|  | Female | 6(33.3) | 4(36.4) | 3(33.3) | 12(48) |
| Decompression of hydronephrosis (%) | | 5(33.3) | 1(10) | 0 | 0 |
| Odinopoeia (%) | | 2(13.3) | 0 | 0 | 0 |
| Postnatal obstruction (%) | | 9(60) | 7(70) | 2(20) | 0 |
| Surgical | | 2 | 2 | 0 | 0 |

**Supplemental Table 2.** Detailed ELISA and US results or follow-ups of every ANH pregnant woman in our research(n=34).

| Sample number | Moesin expression by ELISA (ng/ml) | ANH unilateral/  bilateral | Second trimester US | | | | | Third trimester US | | | | | Decompression of hydronephrosis (times) | US after birth |
| --- | --- | --- | --- | --- | --- | --- | --- | --- | --- | --- | --- | --- | --- | --- |
|  |  |  | Grading system | Ureteral dilatation/obstruction | Parenchymal thinning(mm) | APD2 (mm) | Severity degree by APD | Grading system | Ureteral dilatation/obstruction | Parenchymal thinning(mm) | APD3 (mm) | Severity degree by APD |  |  |
| N1 | 5.32 | left | III | obstruction | 2 | 9 | moderate | IV | obstruction | 2.3 | 17 | severe |  | nonobstructive |
| N2 | 7.76 | bilateral | IV | obstruction | 2.9 | 19 | severe | IV | obstruction | 2.9 | 19 | severe | 1 | obstructive  (Surgical) |
| N3 | 3.26 | left | IV | both | 1.5 | 16 | severe |  |  |  |  |  |  | obstructive  (odinopoeia) |
| N4 | 12.18 | bilateral | IV | both | 2 | 16 | severe | IV | both | 1.7 | 19 | severe |  | obstructive  (odinopoeia) |
| N5 | 8.76 | left | IV | both | 1.6 | 7.8 | moderate | IV | both | 1.5 | 17.6 | severe |  | obstructive |
| N6 | 3.69 | right | III | both | 4.2 | 13 | severe | IV | both | 4.3 | 18 | severe |  | nonobstructive |
| N7 | 5.55 | left | III | obstruction | 4.5 | 11 | severe | IV | obstruction | 6 | 15 | severe |  | obstructive |
| N8 | 7.04 | bilateral | III |  | 4 | 4.2 | mild | IV |  | 3.5 | 8.4 | moderate |  | nonobstructive |
| N9 | 6.67 | bilateral | III | obstruction | 4.1 | 13 | severe | IV | obstruction | 3.9 | 16 | severe |  | obstructive |
| N10 | 8.07 | left | III | both | 3 | 14 | severe | IV | both | 2.3 | 18 | severe | 3 | obstructive |
| N11 | 6.08 | bilateral | III |  | 4 | 13 | severe | IV |  | 4 | 13 | severe |  | obstructive |
| N12 | 4.71 | right | IV |  | 1.6 | 23.7 | severe | IV |  | 1.6 | 23.7 | severe |  | nonobstructive |
| N13 | 8.04 | left | IV | obstruction | 2.3 | 32 | severe | IV | obstruction | 2.3 | 32 | severe | 2 | obstructive |
| N14 | 9.49 | left | IV | both | 2.4 | 20 | severe | IV | both | 2.1 | 51 | severe | 2 | obstructive  (Surgical) |
| N15 | 10.45 | bilateral | III |  | 4.1 | 14.6 | severe | IV | obstruction | 2.5 | 24 | severe | 1 | obstructive  (Surgical) |
| N16 | 8.59 | left | II | obstruction | 4.5 | 7.7 | moderate | IV | obstruction | 5.5 | 10 | severe |  | obstructive |
| N17 | 6.30 | left | II |  | 4.7 | 7.6 | moderate | II |  | 5.3 | 13 | severe |  | obstructive |
| N18 | 4.13 | right | II | both | 5.5 | 7 | moderate | II | obstruction | 4.3 | 8.8 | moderate |  | nonobstructive |
| N19 | 4.56 | left | II | obstruction | 4.9 | 13 | severe | IV | obstruction | 2.7 | 16.9 | severe |  | nonobstructive |
| N20 | 4.45 | left | II | both | 4.3 | 7.9 | moderate | II | both | 4.5 | 8.2 | moderate |  | obstructive |
| N21 | 9.84 | bilateral | II |  |  |  |  | II | both | 5 | 11 | severe |  | obstructive |
| N22 | 8.53 | bilateral | II |  | 4.1 | 8.4 | Moderate | III | both | 4.1 | 10 | severe |  | obstructive |
| N23 | 3.95 | bilateral | II |  | 3.9 | 5.5 | mild | II | obstruction | 4 | 10.6 | severe |  | nonobstructive |
| N24 | 6.16 | left | II | both | 3.1 | 9.7 | moderate | III | both | 2.7 | 12.9 | severe |  | obstructive |
| N25 | 6.92 | bilateral | II |  | 3.9 | 10.8 | severe | IV | obstruction | 2.8 | 17 | severe | 1 | obstructive  (Surgical) |
| N26 | 3.50 | left | I |  |  | 3.3 | mild | I |  |  | 5.8 | mild |  | nonobstructive |
| N27 | 3.66 | bilateral | I |  |  | 4 | mild | I |  |  | 6.2 | mild |  | nonobstructive |
| N28 | 4.91 | left | I |  |  | 5 | mild | I |  |  | 7.7 | moderate |  | nonobstructive |
| N29 | 5.94 | left | I |  |  | 8.4 | moderate | III |  |  | 14 | severe |  | obstructive |
| N30 | 6.49 | bilateral | I |  |  | 5.4 | mild | III | both | 4 | 12.7 | severe |  | obstructive |
| N31 | 5.26 | left | I |  |  | 4.2 | mild | I |  |  | 7.2 | moderate |  | nonobstructive |
| N32 | 3.37 | right | I |  |  | 4.5 | mild | I |  |  | 5.4 | mild |  | nonobstructive |
| N33 | 3.16 | left | I |  |  | 5.8 | mild | III | obstruction | 3.7 | 16 | severe |  | nonobstructive |
| N34 | 3.43 | bilateral | I |  |  | 4.4 | mild | I |  |  | 5 | mild |  | nonobstructive |

| **Supplemental Table 3.** List of all significant proteins identified between ANH-sup and CON-sup using mass spectrometry. (P value<0.05, \|log2FC\|>1) | | | | | | | | |
| --- | --- | --- | --- | --- | --- | --- | --- | --- |
| **protein ID** | **P Value** | **log2FC** | **regulated** |  | **protein ID** | **P Value** | **log2FC** | **regulated** |
| BPIB3 | 0.000664 | 29.48219 | up |  | SVEP1 | 0.000571 | -30.2178 | down |
| BPIB2 | 0.000826 | 28.70128 | up |  | COIA1 | 0.000843 | -28.6621 | down |
| SYPL1 | 0.000805 | 28.6778 | up |  | LOXL3 | 0.00089 | -28.6008 | down |
| PIP | 0.00093 | 28.56964 | up |  | SC5AC | 0.000839 | -28.5788 | down |
| GDPD3 | 0.000838 | 28.56564 | up |  | WNT5B | 0.0009 | -28.2883 | down |
| LEG7 | 0.000888 | 28.43373 | up |  | MMP23 | 0.001004 | -27.899 | down |
| VDAC1 | 0.001042 | 27.85302 | up |  | P4HA1 | 0.001013 | -27.8935 | down |
| K2C5 | 0.00112 | 27.77663 | up |  | PLB1 | 0.001158 | -27.8572 | down |
| PPB1;PPBN | 0.00109 | 27.67857 | up |  | FAM3C | 0.001111 | -27.5458 | down |
| SAP | 0.001137 | 27.58533 | up |  | CALL4 | 0.001154 | -27.4243 | down |
| TMEDA | 0.001255 | 27.08761 | up |  | CO5A2 | 0.001308 | -27.0953 | down |
| MMRN2 | 0.001284 | 27.02561 | up |  | LIS1 | 0.001279 | -27.0126 | down |
| CSK21;CSK23 | 0.001487 | 26.43254 | up |  | RAB13 | 0.001332 | -26.8629 | down |
| AGRF5 | 0.001637 | 26.16805 | up |  | METRL | 0.001333 | -26.8501 | down |
| P85A | 0.001767 | 25.75077 | up |  | SEPP1 | 0.001365 | -26.7629 | down |
| MUC7 | 0.025719 | 23.08068 | up |  | HEPS | 0.001388 | -26.7392 | down |
| PTN13 | 0.038914 | 20.42792 | up |  | GDF6 | 0.001399 | -26.6885 | down |
| SFTPD | 0.04892 | 19.97573 | up |  | CO6A2 | 0.001556 | -26.3338 | down |
| CLCA4 | 0.044396 | 19.72764 | up |  | PNCB | 0.001602 | -26.1378 | down |
| BPIB4 | 0.000197 | 4.357566 | up |  | CHP2 | 0.001647 | -26.0785 | down |
| LOX12 | 0.002473 | 4.083041 | up |  | PTPRH | 0.00176 | -25.7872 | down |
| PSPB | 0.006982 | 3.409585 | up |  | SPB9 | 0.001766 | -25.7609 | down |
| UROM | 0.000872 | 3.405849 | up |  | CLC11 | 0.00181 | -25.6611 | down |
| K2C1 | 0.015656 | 3.011461 | up |  | VATE1 | 0.001822 | -25.6393 | down |
| EPIPL | 0.041191 | 2.851772 | up |  | ATP9A | 0.001984 | -25.3146 | down |
| APOD | 0.027628 | 2.850154 | up |  | EXTL2 | 0.002006 | -25.2796 | down |
| MYOF | 0.012798 | 2.813539 | up |  | CSPG4 | 0.046686 | -20.1758 | down |
| BPIA1 | 0.047804 | 2.798008 | up |  | COFA1 | 0.012599 | -4.27056 | down |
| ICAM1 | 0.004369 | 2.784573 | up |  | FBN2 | 0.006685 | -4.03731 | down |
| K2C78 | 0.012198 | 2.749118 | up |  | LAMA2 | 0.03012 | -3.90876 | down |
| K2C4 | 0.000907 | 2.657098 | up |  | F151A | 0.037141 | -3.5672 | down |
| NPT2B | 0.002849 | 2.288302 | up |  | CLCA1 | 0.033438 | -3.27783 | down |
| TRFE | 0.045086 | 2.248282 | up |  | PEDF | 0.006223 | -3.24638 | down |
| SG3A2 | 0.009349 | 2.042227 | up |  | VILI | 0.021172 | -3.16084 | down |
| APOL1 | 0.007572 | 1.780856 | up |  | DPEP1 | 0.008571 | -3.16004 | down |
| SPB5 | 0.049135 | 1.77956 | up |  | LRP1 | 0.015442 | -3.06649 | down |
| SARG | 0.017945 | 1.759803 | up |  | PGBM | 0.006239 | -2.97053 | down |
| MUC5B | 0.00349 | 1.744699 | up |  | NID1 | 0.016739 | -2.91001 | down |
| APOA2 | 0.010419 | 1.709303 | up |  | IL1AP | 0.048757 | -2.78959 | down |
| BPIB1 | 0.037102 | 1.695677 | up |  | CO1A2 | 0.017679 | -2.77604 | down |
| MOES | 0.007567 | 1.639286 | up |  | APOB | 0.028089 | -2.75484 | down |
| STEA4 | 0.043135 | 1.615092 | up |  | ANX13 | 0.024602 | -2.72649 | down |
| SBP1 | 0.012732 | 1.577828 | up |  | OLFL3 | 0.032421 | -2.46091 | down |
| IST1 | 0.004696 | 1.399714 | up |  | AMPN | 0.023143 | -2.30579 | down |
| SUSD2 | 0.023218 | 1.381434 | up |  | GGT1;GGT3;GGT2 | 0.012762 | -2.13527 | down |
| K2C8 | 0.046516 | 1.378298 | up |  | PLOD1 | 0.024844 | -1.96928 | down |
| ES8L2 | 0.034834 | 1.360152 | up |  | ITIH1 | 0.018297 | -1.92182 | down |
| AL3B1 | 0.003626 | 1.35234 | up |  | LEG4 | 0.028346 | -1.91001 | down |
| PDC10 | 0.046684 | 1.315522 | up |  | FETA | 0.019475 | -1.85482 | down |
| RAI3 | 0.014858 | 1.263813 | up |  | CD14 | 0.030973 | -1.78709 | down |
| FOLR1 | 0.026581 | 1.232434 | up |  | CO1A1 | 0.02632 | -1.63598 | down |
| A2ML1 | 0.040906 | 1.143347 | up |  | CUBN | 0.037438 | -1.61927 | down |
| ANXA5 | 0.008694 | 1.110762 | up |  | FBLN1 | 0.048836 | -1.57836 | down |
|  |  |  |  |  | MA1A1 | 0.026313 | -1.57728 | down |
|  |  |  |  |  | C1QB | 0.039394 | -1.24814 | down |
|  |  |  |  |  | C1QC | 0.026701 | -1.23469 | down |
|  |  |  |  |  | APOE | 0.006922 | -1.20259 | down |
|  |  |  |  |  | GTR1 | 0.042522 | -1.01516 | down |

| Supplemental Table 4 List of all significant mRNAs identified between ANH-sup and CON-sup using RNA sequencing. (P value<0.05, \|log2FC\|>1) | | | | | | | | |
| --- | --- | --- | --- | --- | --- | --- | --- | --- |
| gene ID | Pvalue | log2FC | regulated |  | gene ID | Pvalue | log2FC | regulated |
| OPRPN | 5.31E-16 | 13.2122 | up |  | TTR | 0.00028 | -5.39886 | down |
| STATH | 1.75E-05 | 7.253827 | up |  | FABP2 | 4.05E-08 | -5.00725 | down |
| RPTN | 1.01E-11 | 6.898063 | up |  | CDX1 | 1.41E-05 | -4.94204 | down |
| COL4A3 | 3.93E-09 | 5.384827 | up |  | LGALS2 | 1.08E-08 | -4.5165 | down |
| RTKN2 | 2.96E-16 | 4.768979 | up |  | RBP2 | 7.96E-10 | -4.30397 | down |
| SFTPC | 5.77E-06 | 4.394532 | up |  | CHP2 | 7.28E-17 | -4.13488 | down |
| TFF2 | 0.001688 | 4.348671 | up |  | MUC13 | 7.25E-08 | -4.07234 | down |
| AQP5 | 5.85E-13 | 4.25881 | up |  | SLC7A7 | 2.51E-11 | -4.02999 | down |
| MUC7 | 7.13E-08 | 4.252265 | up |  | USH1C | 0.00045 | -4 | down |
| ASPRV1 | 1.65E-09 | 4.229984 | up |  | SERPINA1 | 5.06E-18 | -3.98543 | down |
| FILIP1 | 2.34E-18 | 4.100149 | up |  | ANXA13 | 0.000443 | -3.9146 | down |
| NKX3-1 | 8.82E-07 | 3.746458 | up |  | APOC3 | 6.21E-08 | -3.78097 | down |
| LYZ | 2.50E-06 | 3.660207 | up |  | ANPEP | 7.03E-15 | -3.73651 | down |
| COL4A2 | 1.53E-08 | 3.24872 | up |  | APOA1 | 8.21E-09 | -3.52609 | down |
| PRR9 | 7.36E-07 | 3.169841 | up |  | G0S2 | 1.17E-06 | -3.50012 | down |
| SLC34A2 | 1.18E-05 | 3.118267 | up |  | FABP1 | 3.73E-06 | -3.47746 | down |
| SCGB1A1 | 0.001365 | 3.015133 | up |  | ALDOB | 1.43E-17 | -3.43721 | down |
| ZG16B | 0.035004 | 2.985793 | up |  | FBXO2 | 1.22E-13 | -3.33568 | down |
| EDN3 | 3.24E-08 | 2.968121 | up |  | TGFBI | 3.33E-08 | -3.31634 | down |
| LRRK2 | 5.47E-05 | 2.661937 | up |  | PHGR1 | 4.75E-08 | -3.2497 | down |
| C9orf24 | 0.033284 | 2.563949 | up |  | SELENOP | 7.09E-09 | -3.22374 | down |
| FILIP1L | 9.31E-05 | 2.500087 | up |  | APOA4 | 1.99E-08 | -2.95413 | down |
| MSN | 4.62E-06 | 2.324026 | up |  | XAGE2 | 1.01E-07 | -2.86668 | down |
| CA11 | 0.000515 | 2.278647 | up |  | EPSTI1 | 6.50E-09 | -2.78103 | down |
| TLCD2 | 5.29E-06 | 2.197179 | up |  | TGM2 | 2.77E-07 | -2.73203 | down |
| RASSF3 | 5.05E-06 | 2.183209 | up |  | NLRP10 | 0.006205 | -2.66321 | down |
| MUC5AC | 0.000657 | 2.181136 | up |  | TNNT1 | 6.57E-07 | -2.66064 | down |
| C20orf85 | 3.42E-05 | 2.180683 | up |  | GBP2 | 1.04E-06 | -2.62903 | down |
| TFF1 | 0.009555 | 2.171598 | up |  | L1TD1 | 2.46E-09 | -2.60668 | down |
| DENND3 | 9.61E-05 | 2.156503 | up |  | COL3A1 | 3.22E-09 | -2.58902 | down |
| SNX31 | 0.00032 | 2.136377 | up |  | KRT1 | 0.001865 | -2.55885 | down |
| AC079447.1 | 2.67E-05 | 2.125351 | up |  | AL645922.1 | 1.71E-05 | -2.55289 | down |
| ITGA3 | 0.00013 | 2.122291 | up |  | COL5A1 | 1.41E-08 | -2.49764 | down |
| NFIX | 1.36E-06 | 2.094209 | up |  | LGALS4 | 0.000438 | -2.47174 | down |
| FOXQ1 | 1.76E-08 | 2.091456 | up |  | TNNT3 | 8.71E-11 | -2.45543 | down |
| CA2 | 0.001068 | 2.088502 | up |  | FN1 | 6.27E-10 | -2.4403 | down |
| GSAP | 2.82E-06 | 2.04265 | up |  | ESPN | 1.25E-06 | -2.41459 | down |
| PALM3 | 5.18E-06 | 2.022786 | up |  | CIB2 | 5.05E-06 | -2.37538 | down |
| AHCYL2 | 9.38E-05 | 1.999351 | up |  | CD68 | 3.56E-10 | -2.36437 | down |
| NDNF | 0.000214 | 1.963028 | up |  | TYROBP | 0.04021 | -2.30048 | down |
| CEP112 | 2.15E-06 | 1.913976 | up |  | IFITM1 | 0.000177 | -2.27371 | down |
| RNF103-CHMP3 | 5.82E-07 | 1.897286 | up |  | FXYD5 | 2.65E-08 | -2.20516 | down |
| AQP4 | 4.55E-07 | 1.836475 | up |  | BCAT1 | 6.55E-07 | -2.18906 | down |
| AKAP12 | 3.86E-05 | 1.832721 | up |  | FSCN1 | 3.13E-05 | -2.17253 | down |
| FBXW4 | 4.09E-05 | 1.812015 | up |  | REEP6 | 7.80E-06 | -2.16741 | down |
| TGFB3 | 0.000249 | 1.805485 | up |  | COL17A1 | 9.54E-07 | -2.13959 | down |
| TNNC1 | 0.029918 | 1.782929 | up |  | KRT17 | 2.51E-07 | -2.11827 | down |
| NCKAP5 | 0.000218 | 1.780957 | up |  | COL1A1 | 1.84E-05 | -2.11213 | down |
| DLC1 | 0.000226 | 1.765772 | up |  | RPL17-C18orf32 | 0.03889 | -2.10538 | down |
| DHRS2 | 0.000256 | 1.732409 | up |  | GSTA2 | 0.003382 | -2.10175 | down |
| TNS1 | 2.30E-05 | 1.712864 | up |  | PDZRN3 | 6.58E-08 | -2.09626 | down |
| SPRR2G | 0.026924 | 1.706397 | up |  | MFAP2 | 0.004508 | -2.08637 | down |
| SFTA3 | 2.36E-06 | 1.703373 | up |  | PDLIM4 | 1.94E-06 | -2.07911 | down |
| VEGFA | 3.36E-05 | 1.702814 | up |  | HTRA3 | 0.001649 | -2.0532 | down |
| GCNT2 | 0.000121 | 1.702306 | up |  | DSC3 | 6.53E-07 | -2.05178 | down |
| MUC5B | 0.006614 | 1.696454 | up |  | INS-IGF2 | 0.003268 | -2.04509 | down |
| FOLR1 | 0.012547 | 1.69624 | up |  | ANXA8 | 2.38E-05 | -2.03814 | down |
| BCL2L2 | 0.003013 | 1.694105 | up |  | HAND1 | 6.06E-05 | -2.02557 | down |
| EFCAB2 | 0.001497 | 1.69191 | up |  | FABP6 | 0.037198 | -1.95972 | down |
| SYDE2 | 1.38E-06 | 1.690447 | up |  | AFAP1L2 | 7.11E-06 | -1.9035 | down |
| FAM184A | 0.000273 | 1.688764 | up |  | FN3K | 1.66E-06 | -1.8935 | down |
| KLC1 | 0.000897 | 1.688218 | up |  | SNAI2 | 3.52E-05 | -1.88905 | down |
| SELENBP1 | 7.26E-05 | 1.685995 | up |  | QPRT | 0.000169 | -1.88414 | down |
| VEPH1 | 4.01E-05 | 1.661369 | up |  | SFN | 5.56E-06 | -1.87315 | down |
| FOXP1 | 3.16E-05 | 1.654288 | up |  | UPK3BL1 | 0.016494 | -1.83666 | down |
| ADGRF5 | 5.67E-05 | 1.651919 | up |  | RGS10 | 0.000205 | -1.82301 | down |
| MTURN | 7.33E-05 | 1.644574 | up |  | CALD1 | 2.85E-06 | -1.81848 | down |
| NFYB | 3.75E-05 | 1.642871 | up |  | CRABP2 | 5.00E-05 | -1.81799 | down |
| ADAMTSL1 | 0.000352 | 1.626418 | up |  | NGEF | 0.001993 | -1.81626 | down |
| ERICH2 | 0.001197 | 1.620576 | up |  | FHL1 | 0.004897 | -1.81316 | down |
| CAT | 0.000255 | 1.61681 | up |  | CCND2 | 1.10E-05 | -1.80458 | down |
| PEX5 | 6.71E-05 | 1.612753 | up |  | BOK | 3.08E-06 | -1.79889 | down |
| ZNHIT6 | 0.00035 | 1.612612 | up |  | ANXA8L1 | 2.80E-05 | -1.77443 | down |
| CGNL1 | 2.92E-05 | 1.609553 | up |  | GCHFR | 0.004983 | -1.77305 | down |
| ARHGAP44 | 0.000342 | 1.607971 | up |  | ID2 | 3.80E-06 | -1.74001 | down |
| AGER | 5.55E-05 | 1.604017 | up |  | CENPE | 5.96E-06 | -1.73402 | down |
| SFTPB | 7.23E-05 | 1.584985 | up |  | ASS1 | 3.61E-07 | -1.7326 | down |
| AC093512.2 | 0.027919 | 1.584956 | up |  | CPXM2 | 1.35E-05 | -1.72753 | down |
| ETV5 | 0.017003 | 1.58412 | up |  | KIF15 | 3.67E-05 | -1.72031 | down |
| FMO2 | 4.02E-05 | 1.573077 | up |  | IGFBP3 | 0.002875 | -1.69782 | down |
| SYNPO | 0.00012 | 1.572008 | up |  | LGALS1 | 0.015473 | -1.69627 | down |
| CTXN1 | 0.007718 | 1.557038 | up |  | MANBAL | 0.002158 | -1.68492 | down |
| FLG2 | 0.000181 | 1.545871 | up |  | OSR1 | 0.003054 | -1.67016 | down |
| FCGR2A | 0.009614 | 1.543097 | up |  | PBLD | 0.000235 | -1.66818 | down |
| CD44 | 3.86E-05 | 1.531746 | up |  | LCP1 | 0.028827 | -1.66536 | down |
| HPS5 | 0.000686 | 1.529203 | up |  | MYADM | 0.00072 | -1.66527 | down |
| KCTD12 | 0.001353 | 1.517393 | up |  | GYPC | 0.002479 | -1.65379 | down |
| AARD | 0.002387 | 1.516072 | up |  | AHNAK2 | 1.17E-05 | -1.65326 | down |
| SESN1 | 0.000131 | 1.514186 | up |  | PTGES | 0.000198 | -1.64981 | down |
| ORMDL3 | 0.001998 | 1.499783 | up |  | RHOC | 1.54E-06 | -1.645 | down |
| CDR1 | 0.001367 | 1.498725 | up |  | SPINK7 | 0.00405 | -1.62946 | down |
| UPK2 | 0.004787 | 1.497596 | up |  | DHRS11 | 0.000631 | -1.62517 | down |
| PRDM5 | 0.000221 | 1.496745 | up |  | TPM1 | 2.32E-05 | -1.60888 | down |
| KIF1C | 0.000176 | 1.475891 | up |  | FYN | 5.53E-05 | -1.59976 | down |
| CAVIN2 | 0.000214 | 1.475665 | up |  | FTL | 2.29E-05 | -1.59298 | down |
| SPINK1 | 0.001428 | 1.472961 | up |  | ZYX | 0.000141 | -1.58896 | down |
| MT-CO3 | 0.044373 | 1.459838 | up |  | DPYSL3 | 6.54E-06 | -1.58859 | down |
| APLP2 | 0.0001 | 1.449165 | up |  | ZFP42 | 0.000323 | -1.58182 | down |
| UPK1A | 0.014736 | 1.447595 | up |  | CAVIN3 | 0.003287 | -1.55765 | down |
| RHCG | 0.017543 | 1.440308 | up |  | GSTA1 | 0.00251 | -1.55366 | down |
| SLPI | 0.006107 | 1.431295 | up |  | SLC2A1 | 0.000169 | -1.54709 | down |
| LIMCH1 | 0.00037 | 1.429323 | up |  | RRAS | 0.000589 | -1.54169 | down |
| IFT57 | 0.00013 | 1.425061 | up |  | CDKN1C | 1.18E-05 | -1.53896 | down |
| WWC2 | 0.000286 | 1.422576 | up |  | S100A4 | 0.000137 | -1.5291 | down |
| TNS3 | 0.000107 | 1.417234 | up |  | OAT | 0.000118 | -1.5284 | down |
| PFKFB3 | 0.000178 | 1.415651 | up |  | FOS | 0.000695 | -1.51973 | down |
| CFAP43 | 0.0003 | 1.412078 | up |  | SMIM1 | 0.02506 | -1.51362 | down |
| ARHGEF26 | 0.000605 | 1.386244 | up |  | STXBP6 | 8.36E-05 | -1.47639 | down |
| SEL1L3 | 0.002683 | 1.371507 | up |  | TCIM | 5.46E-05 | -1.46831 | down |
| NFIC | 0.00047 | 1.371361 | up |  | NNAT | 0.021649 | -1.46366 | down |
| ZSWIM9 | 0.005407 | 1.358421 | up |  | APOBEC3A | 0.030403 | -1.46244 | down |
| MUC1 | 0.002155 | 1.357488 | up |  | H2BC14 | 0.008703 | -1.45414 | down |
| KLHL5 | 0.000569 | 1.339229 | up |  | GABRP | 0.000998 | -1.45241 | down |
| LMO3 | 0.001756 | 1.330641 | up |  | MRPL53 | 0.002997 | -1.45186 | down |
| SMARCA5 | 0.000243 | 1.316966 | up |  | IGFBP4 | 0.001 | -1.44931 | down |
| PTPN13 | 0.000193 | 1.311358 | up |  | SLC40A1 | 0.004626 | -1.44686 | down |
| DAPK2 | 0.000727 | 1.310897 | up |  | SFXN3 | 0.000194 | -1.44234 | down |
| NIBAN1 | 0.000855 | 1.300337 | up |  | VTCN1 | 0.009347 | -1.43641 | down |
| ANKRD37 | 0.043986 | 1.294292 | up |  | PNP | 0.000406 | -1.43141 | down |
| TM4SF1 | 0.009702 | 1.288135 | up |  | HLA-A | 0.003647 | -1.42016 | down |
| ZNF326 | 0.000271 | 1.285708 | up |  | MX1 | 0.004272 | -1.41578 | down |
| NBPF14 | 0.000677 | 1.285425 | up |  | GJB3 | 0.023553 | -1.40137 | down |
| DEDD | 0.007282 | 1.284643 | up |  | PDLIM1 | 7.43E-05 | -1.39949 | down |
| SCAF8 | 0.001916 | 1.276904 | up |  | CIT | 0.000337 | -1.39602 | down |
| CPM | 0.002441 | 1.272397 | up |  | PEPD | 0.000543 | -1.379 | down |
| MYO5C | 0.000411 | 1.267645 | up |  | SLC2A3 | 0.000187 | -1.37541 | down |
| TRIM22 | 0.000461 | 1.266682 | up |  | ADAMTS6 | 0.000366 | -1.37343 | down |
| LAMA5 | 0.018721 | 1.265568 | up |  | FSTL1 | 4.74E-05 | -1.36481 | down |
| PPP2R5A | 0.002002 | 1.260249 | up |  | MDK | 0.001157 | -1.36434 | down |
| SCNN1G | 0.00152 | 1.258132 | up |  | HSPA1A | 0.038249 | -1.36085 | down |
| PLCE1 | 0.000399 | 1.24568 | up |  | EGLN3 | 0.000139 | -1.35467 | down |
| BHMT | 0.015702 | 1.224378 | up |  | IGFBP5 | 0.00542 | -1.35379 | down |
| EPB41L5 | 0.000436 | 1.223468 | up |  | KRT4 | 0.002477 | -1.35327 | down |
| HIRIP3 | 0.015334 | 1.223115 | up |  | HCFC1R1 | 0.000816 | -1.3418 | down |
| NSRP1 | 0.00136 | 1.220193 | up |  | TYMS | 0.012591 | -1.34082 | down |
| PLS1 | 0.000734 | 1.219101 | up |  | SERPINB2 | 0.003074 | -1.33582 | down |
| RIN2 | 0.000525 | 1.215298 | up |  | ARID3A | 0.000148 | -1.33513 | down |
| SHANK2 | 0.000338 | 1.214746 | up |  | ZBED2 | 0.001039 | -1.33426 | down |
| PNKD | 0.003143 | 1.209101 | up |  | VGLL3 | 0.000345 | -1.33414 | down |
| NDUFV3 | 0.00214 | 1.208407 | up |  | PKP3 | 0.000817 | -1.33072 | down |
| TMEM97 | 0.02473 | 1.207095 | up |  | C19orf33 | 0.000209 | -1.33 | down |
| MBIP | 0.001249 | 1.205385 | up |  | SPP1 | 0.021788 | -1.32814 | down |
| SLC9A3R2 | 0.00272 | 1.202463 | up |  | DDIT4 | 0.000613 | -1.32722 | down |
| CASC3 | 0.001393 | 1.198677 | up |  | CERCAM | 0.004157 | -1.32524 | down |
| MICAL2 | 0.024493 | 1.196071 | up |  | S100A10 | 0.000102 | -1.32479 | down |
| MYO1B | 0.002161 | 1.190461 | up |  | RGCC | 0.000272 | -1.32026 | down |
| MTUS1 | 0.000872 | 1.183395 | up |  | ANLN | 0.002578 | -1.3111 | down |
| LAMA3 | 0.013718 | 1.177055 | up |  | SIRT2 | 0.002584 | -1.3094 | down |
| ALDH1A1 | 0.000847 | 1.168262 | up |  | H2AC11 | 0.01049 | -1.30599 | down |
| AC242842.3 | 0.001162 | 1.168212 | up |  | PRR15 | 0.001277 | -1.29701 | down |
| PPA1 | 0.002024 | 1.163098 | up |  | H4C9 | 0.036341 | -1.29472 | down |
| ANP32A | 0.00793 | 1.157136 | up |  | DOK4 | 0.001383 | -1.28401 | down |
| AHR | 0.00058 | 1.156053 | up |  | RHOU | 0.000552 | -1.27082 | down |
| OTUD7B | 0.000785 | 1.154663 | up |  | SPINT2 | 0.002481 | -1.2704 | down |
| BUD13 | 0.004917 | 1.154021 | up |  | TUBB6 | 0.007943 | -1.26463 | down |
| EHBP1 | 0.00128 | 1.147856 | up |  | LMNA | 0.000541 | -1.25591 | down |
| MAP4K5 | 0.004471 | 1.139996 | up |  | H2AC14 | 0.008655 | -1.25388 | down |
| RALGAPA2 | 0.001229 | 1.139041 | up |  | SAT2 | 0.019266 | -1.25319 | down |
| TRIM41 | 0.003106 | 1.128337 | up |  | LDHA | 0.000783 | -1.25176 | down |
| CNN2 | 0.000824 | 1.126987 | up |  | SMIM24 | 0.005498 | -1.25129 | down |
| NBPF10 | 0.004716 | 1.122989 | up |  | PKP1 | 0.000943 | -1.24774 | down |
| RASSF8 | 0.002394 | 1.122071 | up |  | SPARC | 0.000411 | -1.2436 | down |
| PLXNA1 | 0.011222 | 1.119487 | up |  | IGF2BP2 | 0.000205 | -1.24118 | down |
| HNRNPH3 | 0.00293 | 1.114167 | up |  | CRIP1 | 0.000201 | -1.23598 | down |
| SEC63 | 0.000694 | 1.112547 | up |  | S100A6 | 0.000863 | -1.23443 | down |
| TRAK2 | 0.012185 | 1.102107 | up |  | DSC2 | 0.000268 | -1.23172 | down |
| NKX2-1 | 0.000984 | 1.098031 | up |  | GAPDH | 0.000895 | -1.22959 | down |
| SBSN | 0.0311 | 1.092695 | up |  | IGFBP2 | 0.001062 | -1.22501 | down |
| NCOA7 | 0.003849 | 1.090651 | up |  | SLC1A5 | 0.004371 | -1.22455 | down |
| UTRN | 0.005282 | 1.08625 | up |  | TBC1D1 | 0.012653 | -1.22013 | down |
| PGPEP1 | 0.001993 | 1.084493 | up |  | MISP | 0.000651 | -1.21889 | down |
| PARP14 | 0.001754 | 1.083744 | up |  | ASAP1 | 0.00722 | -1.21593 | down |
| GOLIM4 | 0.002503 | 1.080186 | up |  | TFAP2A | 0.003201 | -1.20381 | down |
| SNX14 | 0.022577 | 1.07901 | up |  | ARHGDIB | 0.006971 | -1.20083 | down |
| DNAJC13 | 0.003052 | 1.07547 | up |  | JUN | 0.001687 | -1.19721 | down |
| WDR60 | 0.003035 | 1.071021 | up |  | KRT8 | 0.002113 | -1.19542 | down |
| SNCG | 0.031991 | 1.070369 | up |  | C12orf75 | 0.000928 | -1.18634 | down |
| ANKRD26 | 0.002385 | 1.069191 | up |  | DDAH2 | 0.040175 | -1.18622 | down |
| PPFIBP2 | 0.011722 | 1.06829 | up |  | KRT16 | 0.048997 | -1.17826 | down |
| MAGI3 | 0.007064 | 1.055 | up |  | KLF4 | 0.002102 | -1.17694 | down |
| GNPTAB | 0.004939 | 1.05043 | up |  | TMEM45A | 0.014554 | -1.1753 | down |
| SOD2 | 0.011315 | 1.048203 | up |  | ATP2B4 | 0.000748 | -1.175 | down |
| CRB3 | 0.012793 | 1.043031 | up |  | NEDD9 | 0.000671 | -1.17326 | down |
| ZC3H13 | 0.007673 | 1.039602 | up |  | PDLIM2 | 0.00138 | -1.16373 | down |
| PARD3B | 0.002354 | 1.039315 | up |  | SH3D19 | 0.000954 | -1.16268 | down |
| ESF1 | 0.003789 | 1.038722 | up |  | PLEKHA6 | 0.003332 | -1.15944 | down |
| NUDT16 | 0.013502 | 1.037282 | up |  | GSTO1 | 0.021831 | -1.15313 | down |
| DHRS3 | 0.021203 | 1.029319 | up |  | H2BC15 | 0.006801 | -1.15219 | down |
| MECOM | 0.00176 | 1.020499 | up |  | IFITM3 | 0.00612 | -1.15052 | down |
| NBPF26 | 0.009404 | 1.019123 | up |  | EDN1 | 0.015961 | -1.15009 | down |
| CRACR2B | 0.011469 | 1.016004 | up |  | CDC25B | 0.001824 | -1.14306 | down |
| ATP13A4 | 0.004449 | 1.014852 | up |  | TCP11L1 | 0.002675 | -1.14238 | down |
| TEC | 0.00673 | 1.013256 | up |  | S100A13 | 0.001488 | -1.14148 | down |
| UXS1 | 0.004397 | 1.011852 | up |  | TMEM141 | 0.025218 | -1.12881 | down |
| PLOD2 | 0.00449 | 1.007138 | up |  | ARMCX1 | 0.005974 | -1.12403 | down |
| PPP1R9A | 0.002632 | 1.002947 | up |  | KRT10 | 0.030468 | -1.12315 | down |
| THUMPD3 | 0.007829 | 1.002511 | up |  | CARM1 | 0.00229 | -1.11817 | down |
| NUDT3 | 0.004176 | 1.001984 | up |  | WBP1L | 0.003416 | -1.11614 | down |
|  |  |  |  |  | VIM | 0.002355 | -1.10764 | down |
|  |  |  |  |  | PHLDB3 | 0.01391 | -1.10249 | down |
|  |  |  |  |  | SEPTIN11 | 0.004155 | -1.10118 | down |
|  |  |  |  |  | DAPP1 | 0.013213 | -1.10004 | down |
|  |  |  |  |  | LYRM1 | 0.033133 | -1.09934 | down |
|  |  |  |  |  | AC097634.4 | 0.001585 | -1.09822 | down |
|  |  |  |  |  | GNA11 | 0.000874 | -1.09324 | down |
|  |  |  |  |  | H4C13 | 0.012239 | -1.0919 | down |
|  |  |  |  |  | MDFI | 0.011037 | -1.09007 | down |
|  |  |  |  |  | MICALL1 | 0.002976 | -1.08612 | down |
|  |  |  |  |  | AP2S1 | 0.007362 | -1.08543 | down |
|  |  |  |  |  | PLD1 | 0.001366 | -1.08436 | down |
|  |  |  |  |  | LAMB1 | 0.003113 | -1.08392 | down |
|  |  |  |  |  | TUBB | 0.006452 | -1.0817 | down |
|  |  |  |  |  | CDC42EP5 | 0.010116 | -1.08016 | down |
|  |  |  |  |  | PLIN3 | 0.001372 | -1.07866 | down |
|  |  |  |  |  | TIMP1 | 0.049393 | -1.07604 | down |
|  |  |  |  |  | BNIPL | 0.003304 | -1.0739 | down |
|  |  |  |  |  | HIF3A | 0.00311 | -1.07341 | down |
|  |  |  |  |  | TUBB2A | 0.008442 | -1.0712 | down |
|  |  |  |  |  | SLC9A3R1 | 0.001939 | -1.0565 | down |
|  |  |  |  |  | WNT5A | 0.006807 | -1.04868 | down |
|  |  |  |  |  | RHOG | 0.025417 | -1.04832 | down |
|  |  |  |  |  | KRT6C | 0.012046 | -1.04808 | down |
|  |  |  |  |  | HSPA1B | 0.00574 | -1.04694 | down |
|  |  |  |  |  | PXDC1 | 0.004803 | -1.04649 | down |
|  |  |  |  |  | KRT19 | 0.002387 | -1.0311 | down |
|  |  |  |  |  | GSN | 0.005083 | -1.03055 | down |
|  |  |  |  |  | ANXA2 | 0.002967 | -1.02752 | down |
|  |  |  |  |  | COX8A | 0.029041 | -1.0257 | down |
|  |  |  |  |  | SH3BGRL3 | 0.002426 | -1.02273 | down |
|  |  |  |  |  | COX5B | 0.003547 | -1.02066 | down |
|  |  |  |  |  | NENF | 0.007275 | -1.01883 | down |
|  |  |  |  |  | H2AC13 | 0.01914 | -1.01653 | down |
|  |  |  |  |  | EMP3 | 0.047949 | -1.01628 | down |
|  |  |  |  |  | CRYL1 | 0.014491 | -1.01523 | down |
|  |  |  |  |  | CYP26B1 | 0.004575 | -1.01384 | down |
|  |  |  |  |  | NT5C3A | 0.042913 | -1.01292 | down |
|  |  |  |  |  | EPAS1 | 0.004041 | -1.01084 | down |
|  |  |  |  |  | SLC38A1 | 0.008056 | -1.00809 | down |
|  |  |  |  |  | EIF4EBP1 | 0.026842 | -1.00232 | down |

**Supplemental Table 5.** **Genes and proteins with consistent differential expression trends after combined proteomics and mRNA sequencing analysis.**

| mRNA | | | Protein | | | Differential Regulation |
| --- | --- | --- | --- | --- | --- | --- |
| gene ID | P value | log_2_FC | protein ID | P Value | log_2_FC |  |
| ADGRF5 | 0.0000567 | 1.651918876 | AGRF5 | 0.001636815 | 26.16805406 | up |
| SLC34A2 | 0.0000118 | 3.118266875 | NPT2B | 0.002848814 | 2.288301973 | up |
| MUC5B | 0.00661426 | 1.696453966 | MUC5B | 0.003489531 | 1.744699255 | up |
| ALDH3B1 | 0.04230438 | 0.724230672 | AL3B1 | 0.003626014 | 1.352340197 | up |
| SFTPB | 0.0000723 | 1.584985479 | PSPB | 0.006982287 | 3.409585439 | up |
| MSN | 0.00000462 | 2.324025776 | MOES | 0.007566912 | 1.639285668 | up |
| SELENBP1 | 0.0000726 | 1.685994676 | SBP1 | 0.01273156 | 1.577827812 | up |
| MUC7 | 7.13E-08 | 4.252265407 | MUC7 | 0.025719284 | 23.08068152 | up |
| FOLR1 | 0.01254684 | 1.696240103 | FOLR1 | 0.026580672 | 1.232433734 | up |
| PTPN13 | 0.00019259 | 1.311358172 | PTN13 | 0.038913712 | 20.42791676 | up |
| SELENOP | 7.09E-09 | -3.22373789 | SEPP1 | 0.001364678 | -26.76292926 | down |
| CHP2 | 7.28E-17 | -4.13487741 | CHP2 | 0.001646649 | -26.07854117 | down |
| ANPEP | 7.03E-15 | -3.73650986 | AMPN | 0.023142534 | -2.305794885 | down |
| ANXA13 | 0.00044259 | -3.91460352 | ANX13 | 0.024602081 | -2.726485085 | down |
| COL1A1 | 0.0000184 | -2.11212982 | CO1A1 | 0.026319807 | -1.635980896 | down |
| LGALS4 | 0.00043804 | -2.47173629 | LEG4 | 0.028345728 | -1.910009799 | down |
| IGFBP3 | 0.00287472 | -1.69781566 | IBP3 | 0.034956271 | -0.756098012 | down |
| SLC2A1 | 0.000169102 | -1.547090509 | GTR1 | 0.042522269 | -1.015163409 | down |

**Supplemental Figure 1.** Schematic workflow showing the label-free quantitative proteomic analysis and mRNA sequencing of sEVs and cells.


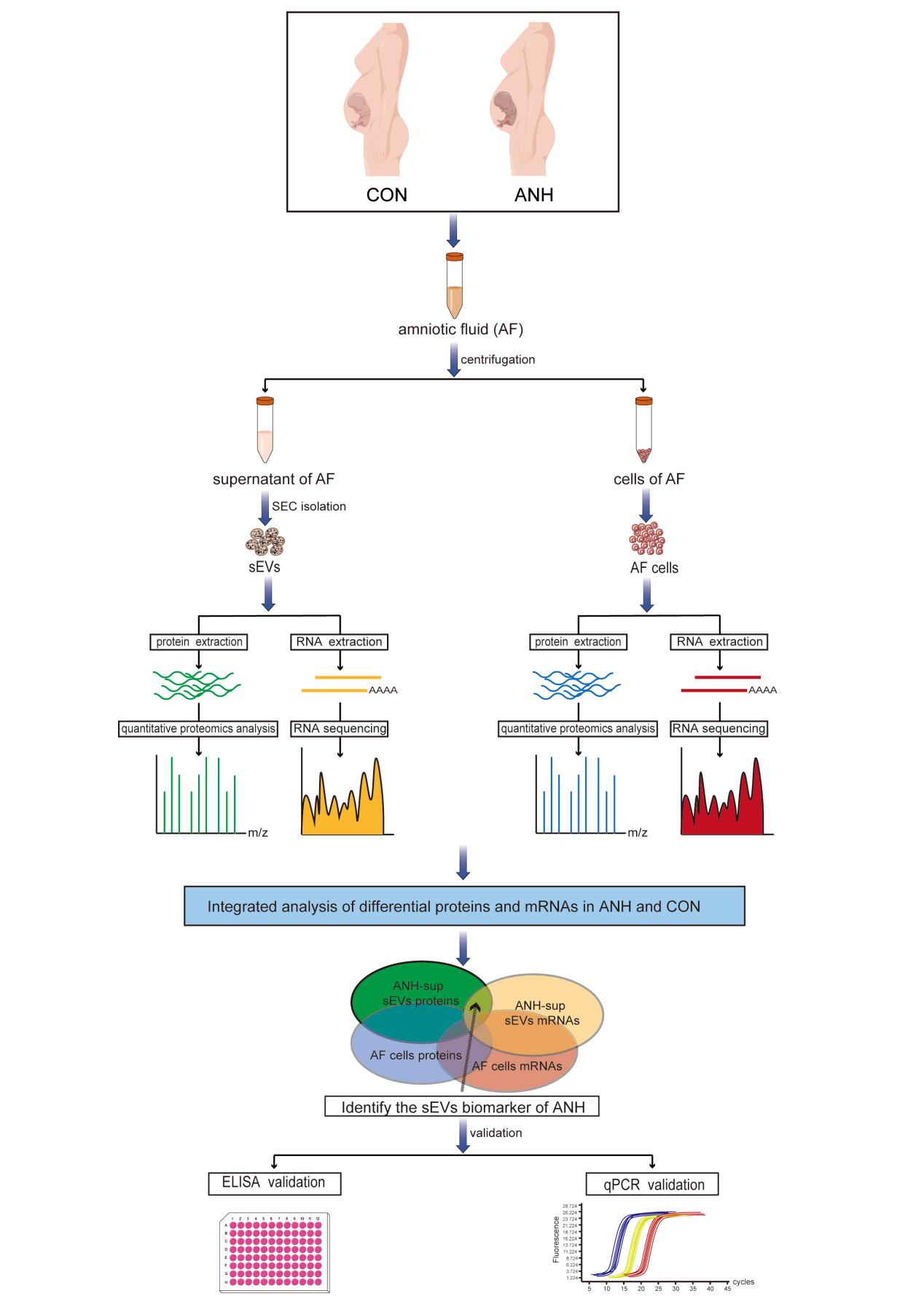


**Supplemental Figure 2.** Target analysis of differentially expressed mRNAs between sEVs and cells in normal fetal groups.


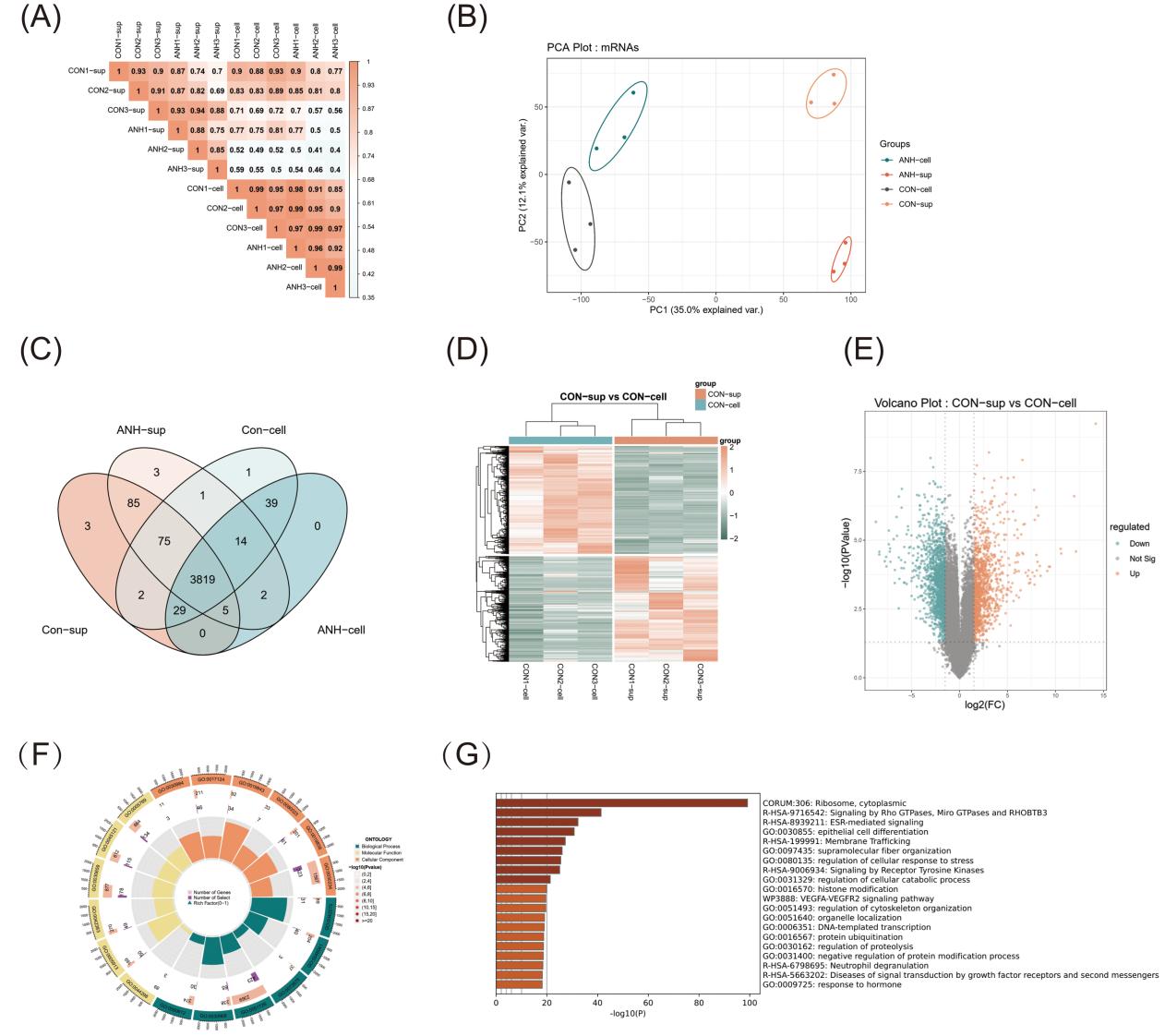


**Supplemental Figure 3.** Validation the expression of candidate Moesin as biomarker.


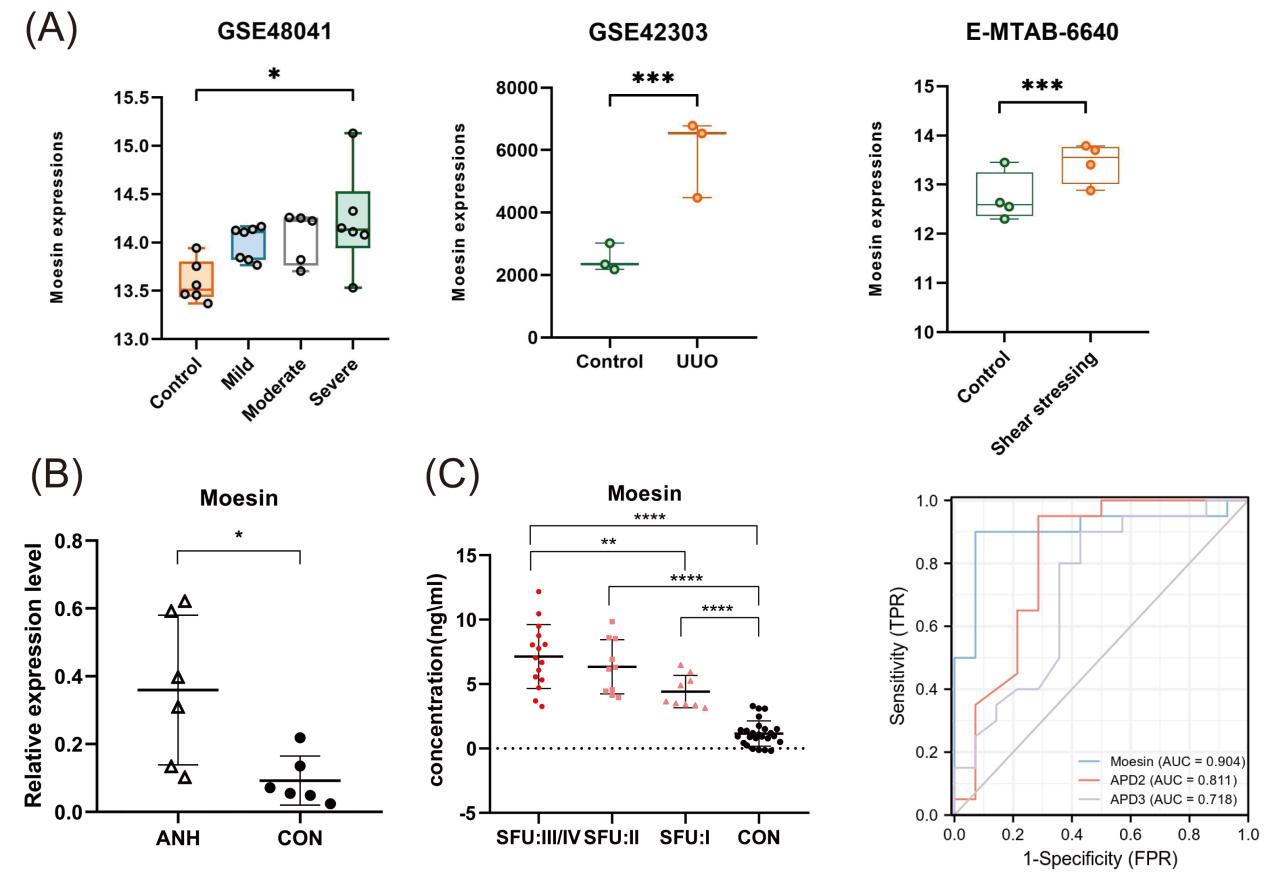

Supplement: Supplementary file 1 — Supporting Information [file CTM2-13-e1360-s001.docx]
